# Neuroprotective Role of Nerve Growth Factor in Hypoxic-Ischemic Brain Injury

**DOI:** 10.3390/brainsci3031013

**Published:** 2013-06-25

**Authors:** Claudia Fantacci, Domenico Capozzi, Pietro Ferrara, Antonio Chiaretti

**Affiliations:** Department of Pediatric Neuroscience, Catholic University of Rome, Largo A. Gemelli 8, Rome 00168, Italy; E-Mails: claudiafantacci@yahoo.it (C.F.); dcapozzi@hotmail.it (D.C.); pferrara@rm.unicatt.it (P.F.)

**Keywords:** neurotrophins, hypoxic-ischemic brain injury, neuroprotection

## Abstract

Hypoxic-ischemic brain injuries (HIBI) in childhood are frequently associated with poor clinical and neurological outcome. Unfortunately, there is currently no effective therapy to restore neuronal loss and to determine substantial clinical improvement. Several neurotrophins, such as Nerve Growth Factor (NGF), Brain-Derived Neurotrophic Factor (BDNF), and Glial Derived Neurotrophic Factor (GDNF), play a key role in the development, differentiation, and survival of the neurons of the peripheral and central nervous system. Experimental animal studies demonstrated their neuroprotective role in HIBI, while only a few studies examined the neuroprotective mechanisms in patients with severe HIBI. We report two cases of children with HIBI and prolonged comatose state who showed a significant improvement after intraventricular NGF administration characterized by amelioration of electroencephalogram (EEG) and cerebral perfusion at single-photon emission computed tomography (SPECT). The improvement in motor and cognitive functions of these children could be related to the neuroprotective role exerted by NGF in residual viable cholinergic neurons, leading to the restoration of neuronal networks in the damaged brain.

## 1. Introduction

Hypoxic-ischemic brain injuries (HIBI) are frequently associated with poor clinical and neurological outcome in survivors, who can suffer from mental retardation, seizures, motor impairment and cerebral palsy [1]. Unfortunately, there is currently no effective therapy which can restore neuronal loss and produce substantial clinical improvement. 

Several neurotrophins have a multifunctional role both in the central and peripheral nervous system [2]. These neurotrophic factors are important regulators of neuronal development, proliferation, differentiation and maturation of the peripheral and central nervous system [3]. Among them, Nerve Growth Factor (NGF), Brain-Derived Neurotrophic Factor (BDNF), Glial cell line-Derived Neurotrophic Factor (GDNF), Neurotrophin-3 (NT3) and Neurotrophin-4 (NT4) seem to play crucial roles in HIBI. Experimental animal models showed that these neurotrophins could be effective in restoring neuronal cells after brain ischemia [4], suggesting that they might be used as therapeutic agents for treating this kind of brain damage [2].

NGF is a neurotrophin which supports the survival and differentiation of neurons during brain development [5]. It has been shown that NGF reduces neural degeneration [6] and promotes peripheral nerve regeneration in rats [7]. It appears globally neuroprotective to the developing brain in a neonatal model of cerebral hypoxia-ischemia [8]. NGF protects against neuronal death and exogenous NGF administration has been shown to prevent or significantly reduce acute cholinergic cell loss and severe neurological deficits following HIBI [9].

It has been reported that also BDNF increases in the cerebrospinal fluid (CSF) of children following HIBI [10]. Intraventricular infusion of BDNF in neonatal rodent models, who received unilateral carotid ligation, resulted in significant protection against both hypoxic-ischemic-induced brain tissue loss and also in spatial memory impairment [11]. GDNF has been shown to have trophic activity on dopaminergic neurons and its endogeneous neuroprotective effect after brain ischemia has been proven [4]. NT3 and NT4 participate in the early development of the brain [12] and have been linked to the survival and functioning of multiple neuronal populations [13]. Furthermore, it is also important to consider that glial cells manufacture neurotrophin receptors and NGF plays a role in normal glial functions [14].

Based on these previous experimental and clinical findings, we report our experience by intraventricular NGF administration in two children suffering from severe HIBI and prolonged comatose state after cardio-respiratory arrest. We hypothesize that NGF infusion after HIBI in pediatric patients could improve neurological outcomes and cerebral perfusion. 

## 2. Experimental Section

The patients, aged 8 and 13 months were admitted to our pediatric intensive care unit (PICU) after prolonged cardio-respiratory arrest and abrupt-onset coma. They were clinically unstable, with a heart rate of about 30 beats/min, small pulses and reduced peripheral perfusion. Mechanical ventilation was started and the haemodynamic stabilization was aimed toward maintaining a normal cerebral perfusion pressure. Because of severe intracranial hypertension, secondary to cerebral edema, the patients underwent external CSF diversion. Persisting severe comatose state and lacking any other feasible therapeutic approach two months after the brain injury, treatment with intraventricular NGF infusion was taken into consideration. A total of 2.5S NGF was purified and lyophilized from male mouse submandibular glands and prepared according to the method of Bocchini and Angeletti [15]. We utilized 1 mg of NGF diluted in 10 ml of saline solution and administered once a day for 10 days consecutively. NGF was infused via the external drainage catheter about two months after the HIBI when the patients continued to exhibit a poor response to conventional and standardized treatment.

The treatment with NGF was approved by the University’s institutional review and ethical board, and by the parents of the infants who provided written informed consent.

**Figure 1:**
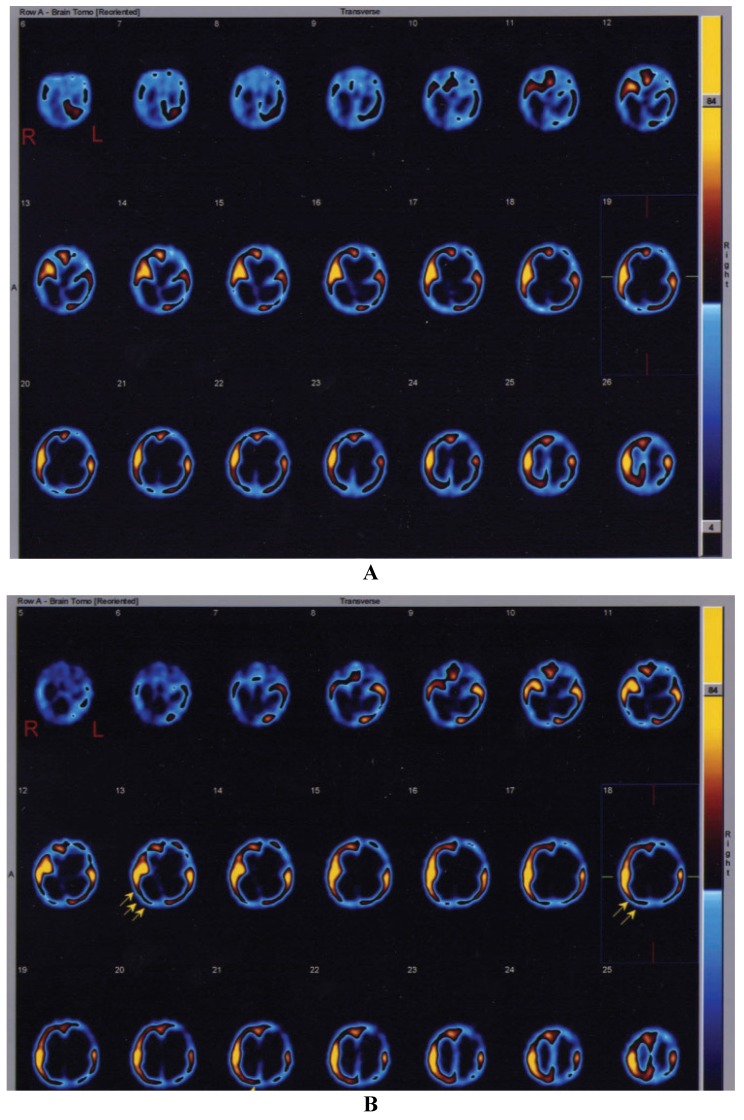
Single-photon emission computed tomography (SPECT) study showing the brain perfusion pattern in the second infant affected by severe hypoxic-ischemic injury. (**A**) Before treatment. (**B**) After intraventricular NGF infusion. The arrows in (**B**) highlight the sites of improvement of cerebral perfusion in right temporal and occipital cortices.

Neurological evaluation before intraventricular NGF infusion revealed a comatose state and asymmetrical tetraparesis with GCS of 4 and 5, respectively. Both the patients presented a global aphasia and no response to environment. After NFG infusion, the infants showed progressive arousal with recovery of awareness, they regained postural control and a significant improvement of limb weakness with spontaneous and finalistic movements; they grasped objects. Moreover, they became expressive with a good level of understanding and able to communicate by significant amelioration of communicative skills. The first child (8 months of age) regained vocalization, the second patient (13 months of age) restarted to pronounce some words he acquired before the cardio-respiratory arrest. Finally, when the patients were discharged from the PICU, one month after the NGF treatment, their GCS scores were 8 and 9, respectively. SPECT images obtained in the first patient before NGF therapy, showed multiple cerebral areas with severe perfusion impairment. In the second SPECT scan carried out one month after NGF treatment, an important improvement of regional cerebral perfusion was visually observed in the right temporal and occipital cortices, with an increase in radiotracer uptake (20.5% and 17.5% in right temporal and occipital regions, respectively). In the second patient, the first SPECT study performed before intraventricular NGF infusion showed absent ^99m^Tc-ECD uptake in left frontal, temporal, parietal and occipital regions. A severe hypoperfusion was also observed in left basal ganglia, right thalamus and left cerebellar hemisphere. One month after NGF treatment, the patient underwent a second SPECT scan, showing a marked increase in radiotracer uptake (21% in right superior frontal area and 90% in right occipital region) (Figure 1A,B).

We did not observe side effects after NGF infusion, such as pain and weight loss. At the two year follow-up, these children are still alive but have some neurological disorders: they have not acquired the equivalent motor and language development as other children of their age, their gait is rather uncoordinated and ataxic, the fine motor skills of their hands are not entirely certain, but they are improving with physiotherapy and speech therapy. 

## 3. Results and Discussion

Previous experimental and clinical studies have shown that HIBI determines an increased expression of NGF and other neurotrophic factors in the central nervous system, and that the NGF up-regulation plays a pivotal role in protecting injured neurons against the biochemical and molecular cascades triggered by ischemic insult [16]. The increased expression of NGF in patients with hypoxic-ischemic brain damage plays a key role in response after injury, and may have a beneficial impact on the regenerative capacity of the injured tissues. Together with NGF, recently other neurotrophins have also been studied concerning their neuroprotective role. BDNF and GDNF are up-regulated at very early stages during brain ischemia, suggesting endogenous neuroprotective mechanisms in viable neuronal networks [17]. Furthermore, exogenous administration of GDNF and BDNF reduces the toxic effects of excitatory amino acids, attenuates nitric oxide production and lowers apoptosis/cell death in stroke animals [4].

Actually, in literature NGF and other neurotrophins are usually associated with the word neuroprotection, even if its use extends well beyond mere neuroprotection and seems to encompass regeneration and neurorehabilitation: this upholds what we have experienced in our clinical practice.

**Figure 2:**
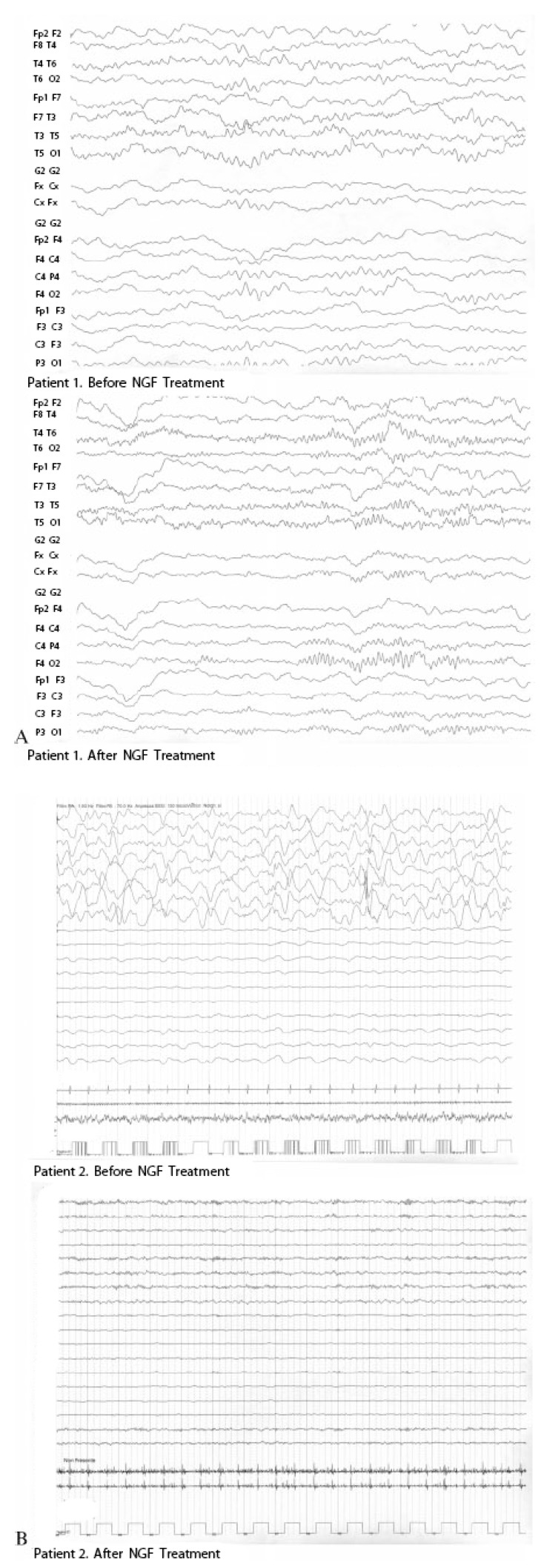
Serial electroencephalogram (EEG) examinations performed before and after the treatment with intraventricular Nerve Growth Factor (NGF) administration showed a constant and progressive reduction in slow-wave activity expressed as an increased alpha/theta ratio in both patients (**A**) patient 1; (**B**) patient 2.

**Figure 3:**
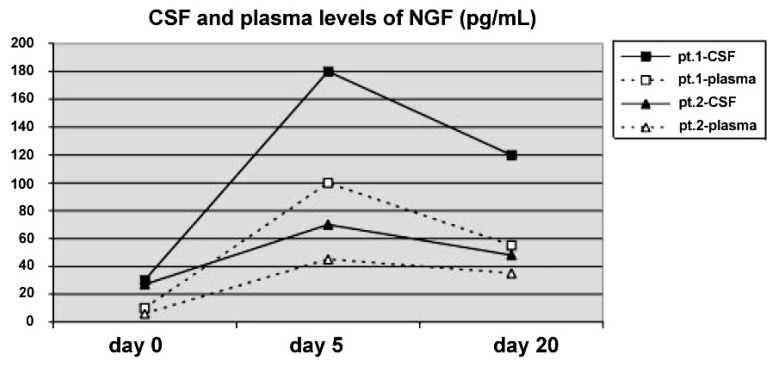
Cerebrospinal fluid (CSF) and plasma levels of NGF (pg/mL) before, during and after the 10 day course of intraventricular NGF infusion in the two infants. The detection limit was 3 pg/mL.

Concerning eventual therapeutic use of neurotrophins in children who suffered from HIBI, our experience provides encouraging results of intraventricular NGF infusion in two infants with prolonged cardio-respiratory arrest, for whom no new therapeutic approaches were proposed other than the conventional treatment. Before NGF administration, severe comatose state, flaccid tetraparesis, and complete aphasia were apparently stabilized in both patients. After the NGF therapy, both infants showed a significant improvement in motor and cognitive functions, with good recovery of their level of awareness, finalistic movements, and amelioration of their communicative skills. The improvement in the state of consciousness and in the communicative functions is congruent with the hypothesis of NGF-induced enhancement of cholinergic brain functions [18]. In these patients, serial EEG recordings showed an important reduction of slow-wave activity after the NGF treatment, and a simultaneous increase in higher frequencies, approximating a more normal EEG pattern (Figure 2). Also SPECT imaging showed a significant improvement of cerebral perfusion related to the concomitant increase of NGF levels in the CSF (Figure 3) [19]. Intraventricular NGF infusion was followed by an improvement of regional cerebral perfusion and selective neurogenesis was demonstrated by the regional 99mTc-ECD uptake increase, reflecting a new cerebral tissue viability and cholinergic functions, as previously reported in literature [20,21]. 

The action of NGF in modulating the cerebral tissue viability might be due to the role exerted by NGF in favoring the biosynthesis of doublecortin (DCX), a protein expressed by new neurons after brain stroke, which indicates a marker for neurogenesis and migrating neuroblasts. In our previous study, we found that intraventricular NGF administration improves the cerebral perfusion and stimulates the pathway of neurogenesis differentiation by the activation of DCX biosynthesis. The DCX increase appears together with the NGF increase in the CSF, raising the possibility that the release of this marker of neuronal precursors may have a pivotal role in neurogenesis mechanisms and neural tissue repair after HIBI [22].

## 4. Conclusions

In conclusion, these observations show that NGF administration might be an effective and safe adjunct therapy in patients with severe HIBI. Moreover, the beneficial effect on nerve function suggests a neuroprotective mechanism exerted by NGF on the residual viable neurological pathways of this subset of patients. Furthermore, other neurotrophins which play crucial roles in brain maturation and development seem to be effective in helping to prevent neuronal loss and brain damage subsequent to HIBI. More prospective and long-term studies are necessary to determine the exact role of neurotrophins as eventual therapeutic agents for injured brain.
